# Complexion and Constitution

**Published:** 1893-09

**Authors:** 


### COMPLEXION AND CONSTITUTION.

Extract from Proceedings of I. H. A.

### Dr. E. E. Case's Paper. Cases of Suppressed Eruption.

DISCUSSION.

Dr. J. H. Allen—I should like to ask Dr. Case why he did not dare to give Sulphur, and in place of it gave Psorinum.

Dr. Case—For the reason that some of our authorities state that Sulphur should not be given after Calcarea. I think Hering makes such a statement in his *Guiding Symptoms.*

Dr. F. O. Pease—What was the complexion of the patient?

Dr. Case—It was stated in the paper.

Dr. F. O. Pease—What method do the best prescribers pursue when they find Calcarea symptoms in a patient with dark hair, black eyes, and sallow skin?

Dr. Case—My own practice is to give Calcarea-phos. in such a case.

Dr. Clark—This is the old idea under another form, of prescribing under some other rule than the law of similia. I am sure that Dr. Lippe used to give Sulphur after Calcarea, and they worked well one after the other.

Dr. Reed—I have long since given up this idea of the sequence of remedies, and of the overwhelming weight of the complexion in determining the selection of a remedy. It might be necessary to give Calcarea to a very scrawny person with dark complexion. The scrawnest patient I ever saw, I prescribed Calc-carb. for, and she is now in the best condition in Boston, although her disease was consumption. There was not a single thing about that patient's build, complexion, or make-up that suggested Calcarea, and yet the symptoms called for it. Psorinum must have been indicated in Dr. Case's patient and not Sulphur. I know it is a very important remedy. Dr. H. C. Allen says that if he had to do away with all remedies except one, he would pick out Psorinum. I have cured eczema with the characteristic brown patches on edges of the scalp, rhagades under the joints with Psorinum when Calcarea, Mercury, or Silicea only palliated, and when Sulphur only relieved for a time.

Dr. H. C. Allen—The simple fact that a patient is over seventy, scrawny and emaciated does not contra-indicate Calcarea, but rather indicates it. We must prescribe according to our law, and these constitutional traits are a part of the symptoms of the case.

Dr. J. H. Allen—I agree with Drs. Allen and Clark in that if a remedy comes under the law of similia prescribe it, no matter what has preceded it. I pay very little attention to what remedy has been given before—or, in other words, to the sequence of remedies.

Dr. Pease—I have found by experience in a few satisfactory cases that it does not always pay to adhere too closely to this question of diathesis under certain remedies. I have in mind one or two cases to which I administered Calc-phos. when the symptoms called for Calc-carb., under the impression that that was the right thing to do when the patients were of dark complexion. It had no effect, while Calc-carb. gave prompt and curative results. I have also a recent case in which Puls. did great good, although the patient was of dark complexion and black hair. The trouble was a chronic one of enlargement of the heart. I hesitated for a long time to give him Puls. on account of his temperament, and tried other remedies. I never had better results than when I gave him Puls.$^{cm}$. He is now improving rapidly.

Dr. Wm. Wesselhœft—When we speak of the diathesis (a word, by the way, which I hate) of a patient, we seek to express in one word his appearance, build, complexion, and temperament. This diathesis should always be regarded as simply one symptom in the case and not anything else. The idea of excluding a remedy because a patient has black hair and is thin, or of prescribing a remedy because the patient is a blond woman and blushes easily is all wrong. It was not the idea of Hahnemann or of his followers. The idea was to give, for the use of the profession, those temperaments in which certain remedies had proved most efficacious or from which the provers had received the most characteristic symptoms. It was not wrong for the gentleman who preceded me to give Pulsatilla to a person of dark complexion with black hair, provided these facts were given due consideration as contra-indications for that remedy. They are to be considered as symptoms, and neither slighted nor given undue weight. In this light it was perfectly legitimate for him to give Pulsatilla. If a fat, fair-haired girl comes into a homœopathic doctor's office and blushes the moment she begins to relate her symptoms he thinks of Calcarea and may ask for more Calcarea symptoms, but it would be wrong to prescribe Calcarea off-hand. Calcarea may be not at all indicated in her case despite her complexion and easy blushing. It would be equally wrong to refuse to consider Calcarea simply because the patient was thin and dark. As regards the sequence of remedies I think there is a great deal to be learned yet. It is a subject upon which we need the observations of our ablest men, not as final or as something which may not be changed, but as helps, capable of further improvement toward the clearing up of a difficult subject. Personally, I am sure that Sulphur does not follow Calcarea well. I am very sure that Rhus does not follow Apis well. I am sure that Sulphur does not follow Lycopodium well. On the other hand, I am sure that Calcarea may follow Sulphur with benefit. I am perfectly convinced that Rhus after Apis has done tremendous harm in erysipelas. I believe if we are thoroughly honest in our observations we may do valuable service to ourselves and to our succesors as to what follows well and what does not follow well.

In regard to the suppression of symptoms which the doctor's paper has so beautifully illustrated for us, I want to give one case from memory. A young lady came to my office with an aggravated case of dyspepsia; she had a sensation as if there was a stone in her stomach, with tremendous depression of spirits and constant eructations of wind, loud and uncontrollable. The attacks would pass off for a time and then come back with the same characteristics. This went on for the better part of a year. One day she came hobbling into my office much better of her dyspepsia, but said she: "Those horrid corns have come back again."

I found that she had had corns for three years; on the little toe were two large, horny excrescences, each one with a seed, as it is called, in the centre; the growth occupied the whole of the little toe. Whenever she had had an attack of corns she had gone to a chiropodist to have them fixed up, and was on her way to his office. She had to go every two or three months; he would shave them down and put Iodine on them and then a blister.

By comparing notes I found that whenever her corns hurt her she was free from gastric symptoms, and that when the chiropodist had cured her corns she had a bad attack of dyspepsia. I told her she must leave her corns to me, and in a new examination, including the corns, I found marked indications for Sepia, a remedy which I had never thought of in her case.

I gave her one dose of Sepia and in less than three months the corns were gone and there was no recurrence of the gastric symptoms; all of which shows that we must respect corns, warts, and every single excrescence which comes on the skin.

Dr. H. C. Allen—Hahnemann lays down a rule in *The Organon* that we are to pay but little attention to any single symptom. We must not prescribe on any single symptom; he also says no single symptom should necessarily throw a remedy out. We are to prescribe on the totality and not on a single symptom. Nor, as I just said, can a single symptom throw out a remedy. While it is true that Nux-vomica, Calcarea, Pulsatilla, Sulphur, Capsicum, and many other remedies have a peculiarly strong effect on people of certain complexions and constitutions, yet it is also true that these remedies *may* be indicated in constitutions the very opposites of those in which they are usually indicated. I have cured perfect Nux-vomica complexions and temperaments with Pulsatilla.

Dr. T. S. Hoyne—Hahnemann also said that we should pay especial attention to those symptoms which are peculiar and characteristic. I think there can be no question but what the father of that child was syphilitic. Between the lines of the essay in which the father was described I can read syphilis. The external manifestation of the disease was suppressed and the child had hereditary syphilis. In children with hereditary syphilis the signs of the disease generally almost always appear during the first four weeks of its existence. If the skin remains clear up to the end of the sixth week we are warranted in excluding congenital syphilis from our diagnosis.

Dr. A. R. Morgan—As I understand the paper it seems to me that the Calcarea 30th and 200th were not used after the manner of Hahnemann.

Dr. E. E. Case—The teachings of Hahnemann are, I believe, to give a remedy and then to wait until the improvement set up gives out, and then either to repeat or give another remedy as the symptoms indicate. At the time I was treating this case I perhaps used the remedies with less skill than I now do. No doubt I gave too much medicine. In regard to Dr. Hoyne's point I could almost take my oath that man never had primary syphilis.

Dr. Hoyne—Still, it is impossible for a man to inherit syphilis and then communicate it as secondary or tertiary syphilis.

Dr. E. E. Case—Could not the active state of the psoriasis in the father, when the child was conceived, have an effect upon the offspring?

Dr. A. R. Morgan—To go back to the original question of Dr. Pease, I should confirm what Drs. H. C. Allen and Wesselhœft have said: the temperament and complexion are suggestive of the remedy, not conclusive. Also, I think most of the failures in treating chronic diseases arise from too frequent prescriptions. The natural inclination is to repeat or change before allowing the full results of the previous dose to take place.

Dr. A. Fisher—Referring to the case of corns reported by Dr. Wesselhœft, I acknowledge that corns are generally a constitutional effect, but I also maintain that very frequently they depend upon bad footgear. The best local treatment I have ever found is to rub them with common yellow soap.

Dr. Holmes—Do you use water also?

Dr. Fisher—No, sir, not at all—and then pull the stockings on over the soap. This softens them by making a lather with the perspiration and makes them painless, and is not, I think, an unjustifiable interference with the action of the indicated remedy whatever that may be.

